# Supplementary material for: Effects of Dietary Protein Source and Quantity on Bone Morphology and Body Composition Following a High-Protein Weight-Loss Diet in a Rat Model for Postmenopausal Obesity
Source: Nutrients. 2022 May 28;14(11):2262. doi: 10.3390/nu14112262 (PMC9183012; doi:10.3390/nu14112262)
Supplement: Supplementary file 1 [file nutrients-14-02262-s001.zip › nutrients-1730948-supplementary.pdf]

Supplemental Table S1: High Fat, High Sugar Diet Formulation[1]

	High Fat, High Sugar (g)
**Protein Source**	
Casein	140
L-Cysteine	1.8
**Carbohydrates**	
Corn Starch	21.5
Maltodextrin 10	125
Sucrose	223
Cellulose	50
**Fat**	
Soybean Oil	40
Lard	146
TBHQ	0.008
**Micronutrients**	
Mineral Mix S10022M	35
Vitamin Mix V10037	10
Choline Bitartrate	2.5
Energy, kcal g	4.6
Protein, % kcal	14
Carbohydrate, % kcal	41
Fat, % kcal	45

[1]TBHQ: tert-Butylhydroquinone, Research Diets Inc: D15053103

Supplemental Table S2. Nutritional Analysis of Freeze-Dried Lean Beef Powder[1]

Amino Acid Profile*		Fatty Acid Profile *(% of Total Fat)*	
Taurine	0.14		
Aspartic Acid	0.26	Myristic (14:0)	2.60
Threonine	7.29	Myristoleic (9c-14:1)	0.73
Serine	3.50	C15:0	0.38
Glutamic Acid	2.75	Palmitic (16:0)	23.37
Proline	12.17	Palmitoleic (9c-16:1)	3.37
Glycine	2.94	Margaric (17:0)	1.17
Alanine	0.00	10c-17:1	0.00
Cysteine	3.57	Stearic (18:0)	11.96
Valine	4.54	Elaidic (9t-18:1)	0.44
Methionine	0.82	Oleic (9c-18:1)	41.31
Isoleucine	4.16	Vaccenic (11c-18:1)	0.00
Leucine	2.13	Linoleic (18:2n6)	3.83
Tyrosine	3.94	Linolenic (18:3n3)	0.18
Phenylalanine	6.57	Stearidonic (18:4n3)	0.00
Ornithine	3.50	Arachidic (20:0)	0.10
Lysine	3.33	Gonodic (20:1n9)	0.71
Histidine	0.12	Homo-a-linolenic(20:3n3)	0.00
Arginine	0.03	Arachidonic [20:4n6]	0.68
Tryptophan	7.19	3n-Arachidonic (20:4n3)	0.04
Total Amino Acid	77.92	EPA (20:5n3)	0.00
		Behenoic (22:0)	0.22
		Erucic [22:1n9]	0.00
Crude Protein*	75.27	Clupanodonic (22:5n3)	0.18
Crude Total Fat	16.94	DHA (22:6n3)	0.02
Percent Fat#	5.6%	Lignoceric (24:0)	0.01
Ash	3.64	Nervonic (24:1n9)	0.00

[1]Grams per 100g Freeze-dried beef; *Percentage Nitrogen X 6.25, includes all nitrogen compounds; #Calculated from the fresh, unfreeze-dried sample

Supplemental Table S3. Main effect of weight loss on bone microarchitecture following 12-weeks of energy restriction[1]

	Baseline	Weight Loss Effect[2]	Diet Effect[2]			
Distal Metaphysis			NP-Control	HP-Beef	HP-Milk	HP-Soy
VcaBMD (mg HA/ccm)	800 ± 10	0.488	0.431	0.569	0.578	0.816
Tb BMC (mg HA x 10^2)	1.94 ± 0.28	**<0.0001**	**<0.0001**	**<0.0001**	**<0.0001**	**<0.0001**
BV/TV x 10^2	23.58 ± 3.71	**<0.0001**	**<0.0001**	**<0.0001**	**<0.0001**	**<0.0001**
Tb.N (mm^{-1})	2.26 ± 0.39	**<0.001**	**<0.001**	**0.001**	**0.003**	**0.015**
Tb.Th (mm)	0.11 ± 0.01	0.859	0.996	0.847	0.989	0.997
Tb.Sp (mm)	0.49 ± 0.08	**0.019**	0.108	**0.009**	0.267	0.597
SMI	1.34 ± 0.25	**0.001**	**0.025**	**<0.001**	**0.005**	0.067
Conn.D $(mm^3)^{-1}$	46.79 ± 10.67	**<0.0001**	**<0.0001**	**<0.0001**	**<0.0001**	**<0.0001**
Midshaft Diaphysis						
VctBMD (mg HA/ccm)	1300 ± 5	0.109	0.999	0.999	0.996	0.449
TA (mm^2)	9.86 ± 0.44	0.164	0.539	0.343	0.679	0.108
Ct.A (mm^2)	6.68 ± 0.30	0.906	0.880	0.995	0.960	0.968
Ct.A/TA	0.68 ± 0.04	0.067	0.119	0.189	0.282	**0.036**
Ma.Ar (mm^2)	3.38 ± 0.34	0.065	0.210	0.189	0.391	**0.033**
Ct.Th (mm)	0.69 ± 0.03	0.179	0.195	0.754	0.340	0.197
pMOI (mm^4)	15.99 ± 1.10	0.786	0.977	0.980	0.908	0.746
Vertebral Body						
VcaBMD (mg HA/ccm)	923 ± 11	0.957	0.954	0.972	0.996	0.998
Tb BMC (mg HA x 10^2)	32.51 ± 16.26	0.128	0.249	0.068	0.348	0.310
BV/TV x 10^2	40.04 ± 5.26	0.106	0.173	0.059	0.359	0.235
Tb.N (mm^{-1})	3.55 ± 0.43	0.328	0.492	0.225	0.458	0.467
Tb.Th (mm)	0.108 ± 0.005	0.328	0.765	0.263	0.925	0.814
Tb.Sp (mm)	0.273 ± 0.035	0.437	0.769	0.317	0.826	0.772
SMI	0.33 ± 0.16	0.162	0.998	0.523	0.998	0.957
Conn.D $(mm^3)^{-1}$	45.51 ± 3.84	0.555	0.936	0.829	0.598	0.550

[1]Mean ± SEM. Conn.D, connectivity density; Ct.A/TA, cortical area fraction; Ct.A, cortical bone area; Ct.Th, cortical thickness; pMOI, polar moment of inertia; Ma.Ar, marrow area; SMI, structural model index; TA, total area; Tb BMC, trabecular BMC; Tb.N, trabecular bone number; Tb.Sp, trabecular bone separation; Tb.Th, trabecular bone thickness; BV/TV; trabecular bone volume fraction; VcaBMD, volumetric cancellous BMD; VctBMD, volumetric cortical BMD

[2]Two-Way ANOVA: Main effect of Weight Loss & Diet. Significance denoted by bolding ($p<0.05$); Post-hoc analysis utilized Tukey's HSD
